# Supplementary material for: ChatGPT-4o can serve as the second rater for data extraction in systematic reviews
Source: PLoS One. 2025 Jan 7;20(1):e0313401. doi: 10.1371/journal.pone.0313401 (PMC11706374; doi:10.1371/journal.pone.0313401)
Supplement: S1 Table — (DOCX) [file pone.0313401.s001.docx]

# Supplementary

**S1 Table: Prompts for ChatGPT-4o.**

List of prompts in each of the five domains of extracted data inserted in ChatGPT-4o.

| Domain | Topic | Prompt |
| --- | --- | --- |
| 1. Baseline information | Year published | Was it reported what year the article was published? What year was the article published? |
|  | Author(s) | Were the names of the authors reported? Please state the names of all authors. |
|  | Protocol registration | Was it reported whether a protocol was published? If so, please state where the protocol was published. |
|  | Funding source | Were any funding sources reported? If yes, how was the study funded? |
|  | Type of trial | Was the study type reported? If yes, what type of study is it? |
|  | In- and exclusion criteria | Were inclusion and exclusion criteria reported? If yes, please state the inclusion and exclusion criteria. |
| 2. Participants baseline information | Setting | Were participant characteristics reported? What was the residential status of the participants? |
|  | Age | Was the age of participants reported? What was the mean or age range of the included participants? |
|  | Gender | Was the gender of participants reported? What was the gender of the participants? |
|  | Falls history (past 12 months) | Was it reported if participants had a fall within the past 12 months prior to the study? If reported, please state the number of participants who had 1 or more falls prior to the study. |
| 3. Participant number | Included participants | Was the number of included participants reported? How many participants were assigned to the intervention and comparator group? |
|  | Drop-outs | Was the number of drop-outs reported? If yes, what was the number of participants who did not complete the intervention? |
| 4. Intervention description | Intervention | Was the intervention training protocol described? If so, what was the training protocol? |
|  | Multiple interventions | Was it reported whether the training protocol was combined with other types of training or interventions? If so, please state the additional intervention. |
|  | Supervised | Was it reported whether training sessions were supervised? Were the training sessions supervised? |
|  | Intervention dose | Was the intervention dose reported? If so, please state the number of training sessions for the intervention and control group. |
|  | Intervention duration | Was the duration of intervention reported? If reported, how long was the intervention period? |
|  | Comparison | Was the protocol for the comparator group reported? If yes, what was the protocol for the comparator or control group? |
| 5. Outcome results | Daily-life-falls | Do the study collect data on daily-life-falls. If yes, how is a daily-life-fall defined, and how are information daily-life-falls collected? |
|  | Daily-life-falls data | If the study collected data on daily-life-fall how many participants provided data for this outcome in the groups, and how many daily-life-falls were reported? |
|  | Laboratory -falls | Does the study collect data on laboratory falls? If yes, how are laboratory falls defined, and how is information on laboratory falls collected? |
|  | Laboratory-falls data | If the study collected data on laboratory falls, how many participants provided data for this outcome in the groups, and how many laboratory falls were reported? |
