# Supplementary material for: ChatGPT-4o can serve as the second rater for data extraction in systematic reviews
Source: PLoS One. 2025 Jan 7;20(1):e0313401. doi: 10.1371/journal.pone.0313401 (PMC11706374; doi:10.1371/journal.pone.0313401)
Supplement: Attachment — Submitted filename: Response to reviewers.docx [file pone.0313401.s004.docx]

Dear Weiqiang (Albert) Jin,

Thank you for considering our manuscript for publication in PLOS ONE and the invitation to resubmission following minor revisions.

We would like to sincerely thank you and the reviewers for your thoughtful feedback and useful suggestions on our manuscript. We have carefully considered all the comments and made the necessary revisions to address the reviewers' concerns.

We believe the manuscript has improved markedly by accommodating the excellent comments. There was a consensus among the reviews that our paper lacked information on the data extraction in ChatGPT-4o, figure and table captions, and discussion points, such as limitations and ethical considerations. We have rewritten parts of the methods and results section, and largely added essential discussion points to accommodate the observations.

Below, we provide a point-by-point response to each comment, explaining how we have incorporated the suggested changes into the revised manuscript.

Sincerely,

On behalf of the coauthors,

Mette Motzfeldt Jensen, MD.

Aalborg University Hospital

Hobrovej 18-22

9000 Aalborg

Denmark

**Editors Comments to Authors:**

Dear authors:
After going through the manuscript and the reviewers' comments, I suggest a Minor Revision (as a associate editor).

The study is promising and offers some great insights into using AI / gpt-4o for data extraction in systematic reviews, but there are a few areas that need a bit more work. The key points to address include adding more details about the methods—like how the sample size was chosen, the specific prompts used, and how the human reviewers did their work. Also, expanding the results and discussion sections to clarify the types of errors found, ways to reduce them, and any potential limitations would help. It's also important to make sure the manuscript follows the proper guidelines for reporting AI research and to polish the language for better readability.

| **Reviewer comment** | **Author reply** | **Location of change in the manuscript** |
| --- | --- | --- |
| 1. Please ensure that your manuscript meets PLOS ONE's style requirements, including those for file naming. The PLOS ONE style templates can be found at <https://journals.plos.org/plosone/s/file?id=wjVg/PLOSOne_formatting_sample_main_body.pdf> and <https://journals.plos.org/plosone/s/file?id=ba62/PLOSOne_formatting_sample_title_authors_affiliations.pdf> | Thank you for your feedback.  We have worked to ensure that the manuscript meets the style requirements. | Throughout the manuscript |
| 2. When completing the data availability statement of the submission form, you indicated that you will make your data available on acceptance. We strongly recommend all authors decide on a data sharing plan before acceptance, as the process can be lengthy and hold up publication timelines. Please note that, though access restrictions are acceptable now, your entire data will need to be made freely accessible if your manuscript is accepted for publication. This policy applies to all data except where public deposition would breach compliance with the protocol approved by your research ethics board. If you are unable to adhere to our open data policy, please kindly revise your statement to explain your reasoning and we will seek the editor's input on an exemption. Please be assured that, once you have provided your new statement, the assessment of your exemption will not hold up the peer review process. | We will make our data publicly available in line with PLOS ONE’s open data policy. We will provide the our datasets as Supplementary. | Supplementary “S2 and S3 Dataset” |
| 3. Please include a separate caption for each figure in your manuscript. | Separate captions for each figure and table have been included. | Methods and results section. |
| 4. Please include captions for your Supporting Information files at the end of your manuscript, and update any in-text citations to match accordingly. Please see our Supporting Information guidelines for more information: <http://journals.plos.org/plosone/s/supporting-information>. | Separate captions for Supporting information have been included to comply with the guidelines. | Supplementary file “S1 Table”. |
| 5. Please review your reference list to ensure that it is complete and correct. If you have cited papers that have been retracted, please include the rationale for doing so in the manuscript text, or remove these references and replace them with relevant current references. Any changes to the reference list should be mentioned in the rebuttal letter that accompanies your revised manuscript. If you need to cite a retracted article, indicate the article’s retracted status in the References list and also include a citation and full reference for the retraction notice. | We have reviewed our reference list to ensure that it is complete and accurate. All changes to the reference list are noted in the revised manuscript. The reviewers’ suggestions of relevant articles have been included (ref 5, 7 and 17). Furthermore, we included ref 14 and 16 which we also found relevant for the manuscript. | Reference list |

**Reviewers' Comments to Author:**

Reviewer: #1

Comments to the Author:

Thank you for submitting your manuscript. I have carefully read through the entire paper and believe this study presents an innovative approach with notable potential for enhancing data extraction processes in systematic reviews. The experimental design is robust, and the results are promising. However, there are a few areas where the manuscript could benefit from minor revisions to further improve clarity and impact. In summary, I recommend minor revision. Below are my specific comments, which I hope will assist you in revising and improving the paper.

| **Reviewer comment** | **Author comment** | **Location of change in the manuscript** |
| --- | --- | --- |
| 1. Insufficient Detail in Background Description: The background section mentions that systematic reviews are a key tool for translating clinical trial evidence into guidelines but does not sufficiently explain why data extraction is a crucial step in systematic reviews and how it impacts the quality of the review. It is recommended to add a discussion on the limitations of current manual data extraction methods and the potential benefits of AI intervention in this section. | Thank you for your feedback.  We have emphasized the critical role of data extraction in systematic reviews in the background section. We also added examples of limitations of manual data extraction. | Introduction, 3^rd^ paragraph. |
| 2. Results Section Needs More Comparative Detail: The results section compares data extraction by ChatGPT-4o with that of human reviewers but does not provide detailed information about the data extraction process used by human reviewers. To enhance the persuasiveness of the comparison, it is suggested to include detailed information about the background and data extraction criteria of human reviewers to ensure fairness and accuracy. Additionally, a description of the human reviewers' workflow could help better understand the differences and advantages between AI and human review processes. | We have made modifications to the results section regarding the human reviewers.  Additionally, a caption for Figure 1 in the methods section have been added, which also may add to a better understanding of the overall study design. | Results. 1^st^ paragraph. |
| 3. Emphasis on Study Limitations in Discussion: While the conclusion affirms the potential of ChatGPT-4o as an auxiliary tool for systematic reviews, the study does not address its limitations, such as the potential for AI to overlook complex contextual relationships or inherent biases. It is recommended to discuss these potential limitations in the discussion section and explore ways to address these issues in future research. | We appreciate your suggestion. We have expanded the discussion to acknowledge limitations of ChatGPT-4o. | Discussion. From line 250. |
| 4. Accuracy of Keywords: The keywords cover the core concepts of the study, but it may be beneficial to include more targeted terms such as “automated data extraction” and “machine learning” to enhance search relevance. | Thank you for your relevant suggestions for more targeted keywords. They have been added. | Title page. Keywords. |
| 5. Data Sources and Representativeness: The study mentions randomly selecting 11 articles to test the capabilities of ChatGPT-4o but does not elaborate on the specifics of the random selection method or the representativeness of the sample. It is recommended to provide more information on how the sample was ensured to be representative, such as whether different types of studies or data reporting quality were considered, to ensure the generalizability of the test results. Incorporating these adjustments will enhance the manuscript's overall quality and provide readers with a clearer understanding of the study's implications and applications. | A random selection procedure was applied, rather than selecting studies based on type or data reporting quality. This approach aimed to ensure diversity among the included studies, encompassing aspects beyond known characteristics.  We have also added to the discussion the need for future studies to explore ChatGPT-4o's performance across more diverse types of studies. | Discussion. From line 279. |

Reviewer: #2

Comments to the Author: This manuscript contributes valuable insights into the use of AI, specifically ChatGPT-4o, for data extraction in systematic reviews. However, there are some points that need further clarification and refinement.

| **Reviewer comment** | **Author comment** | **Location of change in the manuscript** |
| --- | --- | --- |
| 1. External Validity: 1.1 The study's focus on a single ongoing systematic review raises concerns about the generalizability of the findings. Systematic reviews can encompass a wide range of research questions, including interventional, diagnostic, and prognostic studies, etc. It would be beneficial to consider whether the inclusion of different types of studies or systematic reviews might improve the validity and reproducibility of ChatGPT-4o's data extraction capabilities. | Thank you for your feedback. At this stage, we are not able to include additional systematic reviews or types of studies in the current research. Still, we agree that this limitation should be addressed. We added a section in our discussion highlighting this as a potential limitation and emphasize the need for future studies to assess ChatGPT-4o's performance across a broad spectrum of systematic reviews. | Discussion. 1^st^ paragraph in the Strengths and Limitations section. |
| 1.2 The sample size of 11 studies in one systematic review used to evaluate validity is relatively small. This limited sample size may not be sufficient to draw robust conclusions about the general applicability of ChatGPT-4o. Further justification for the adequacy of this sample size would strengthen the manuscript. | While we agree that a larger sample size would provide more robust evidence, we believe that this study serves as an important initial evaluation of ChatGPT-4o’s performance in the context of systematic reviews. To address this concern, we will highlight the perspective of future research to assess ChatGPT-4o’s performance on a larger scale. | Discussion. 1^st^ paragraph in the Strengths and Limitations section. |
| 2. Introduction: 2.1 The Introduction would benefit from a more comprehensive review of existing literature on the use of large language models (LLMs) for data extraction. For example, including references such as DOI: 10.1016/j.ymeth.2024.04.005, among others, would help contextualize the novelty and contributions of the current study. | Thank you for this relevant suggestion. We have incorporated the reference along with other relevant studies in the revised version of the manuscript to provide a broader and more complete review of the field. | Introduction, second paragraph. |
| 3. Methods: 3.1 ChatGPT-4o data extraction:  The manuscript lacks details on the specific prompts used for data extraction with ChatGPT-4o. Providing this information would enhance the transparency and reproducibility of the study. | Thank you for this suggestion to improve transparency.  The specific prompts used for data extraction is provided as supplementary, S1 Table. | Supplementary S1. |
| 3.2 Comparison of data extracted by ChatGPT 4o with the reference standard: It is unclear how the comparison between ChatGPT-4o and human data extraction was conducted. Specifically, were the evaluations conducted by one or two researchers? Clarifying this aspect of the methodology is important for assessing the rigor of the study. | We acknowledge the need for a clearer description of this procedure. We have rewritten the paragraph regarding comparison in the methods section. Furthermore, a caption for Figure 1 which shows the overview of the design has been added, and hopefully adds to the understanding of the design. | Methods. Caption for Figure 1 (line 95) and text from line 119. |
| 3.3 Validity goals and expected utility of ChatGPT as a data extraction tool: Providing a clearer reference to the categories for validity assessment would improve the clarity of the methods. | To our knowledge, no other studies at the time of our study had applied validity goals and categories for the use of AI. The categories were pre-specified by our research team based on what should be considered relevant for assessing the tool’s performance. As this was new grounds, we published these pre-specified categories and validity goals in OSF prior to the conduction of the study to ensure transparency in our methodology. This information has been emphasized in the revised manuscript. | Methods. From line 120. |
| 3.4 The manuscript mentions 22 data points extracted from each study but does not specify what these data points are. Detailing the specific data points would aid readers in understanding the scope of the data extraction process. | The 22 data points mentioned refers to the 22 prompts entered in ChatGPT-4o for each article. The prompts are provided as Supplementary table S1. We have rewritten the section where we mention 22 data points to clarify the data we refer to. | Results. 1^st^ paragraph. |
| 4. Results: 4.1 Consider providing examples of the typical data extraction results in the appendix. This would allow readers to better assess the performance of ChatGPT-4o. | Thank you for your suggestion.  We now provide the data extraction results in the appendix as part of our data sharing, where we make all data available. | Supplementary S2 and S3 Tables (excel sheets). |
| 4.2 The manuscript mentions that 5.2% of the data extracted by ChatGPT-4o was incorrect. A brief discussion of the types of hallucinations observed and potential strategies for identifying or mitigating these errors would be useful. | Thank you for emphasizing this important issue. We agree that a discussion of the types of hallucinations observed in ChatGPT-4o's responses and potential strategies for identifying these errors are valuable. This have been added to the manuscript. | Discussion. From line 229 |
| Other Comments: 5. Adherence to Reporting Guidelines: The manuscript should align with reporting guidelines for AI-related clinical research, such as those mentioned in Flanagin et al. (2024), "Reporting Use of AI in Research and Scholarly Publication-JAMA Network Guidance." Providing details on the prompts used, the time frame of ChatGPT-4o usage, and other relevant methodological aspects would be beneficial. | Thank you for sharing the reporting guidelines for AI-related clinical research.  We have added more details on the prompts used, and time frame of ChatGPT usage to comply with the guidelines. The reference of guidelines have been added. | Prompts are available in Supplementary S1.  The time frame is now provided in Methods section, line 91.  The reference of the reporting guidelines has been added in the discussion below the ethics section. |
| 6. Language and Clarity: The manuscript contains some language that could be further refined for clarity. For example, the sentence "this finding was supported when looking across information domains where agreement was lower for outcome data and a larger proportion of information was not reported" could be simplified for better readability. | Thank you for your helpful feedback. We agree that certain sentences could be refined for better readability. The particular sentence has been revised, and the manuscript reviewed for similar instances to ensure better readability throughout the manuscript. | Results section, line 195, and throughout the manuscript. |

Reviewer: #3

This is an interesting and novel study exploring the use of AI, LLM specifically, in collecting data for systematic reviews. However, I have a few comments that I would like the authors of this study to address.

| **Reviewer comment** | **Author comment** | **Location of change in the manuscript** |
| --- | --- | --- |
| 1. Please provide how you arrived that a sample size of 11 would be adequate to test the validity and reproducibility of ChatGPT 4o. | Thank you for your feedback. We acknowledge that a sample of 11 studies may appear limited for assessing the validity and reproducibility of ChatGPT-4o. However, it is the number of studies times the number of data extracted that describes the robustness and accuracy of our findings. As we provide an early study of AI for support of systematic reviews, our study should also be used to show ways to improve future studies of this kind. We published our pre-specified criteria to support these considerations. This has been highlighted in the revised manuscript.  The selection of 11 studies was based on practical considerations, including the availability of high-quality randomized controlled trials (RCTs) in the specific domain of fall prevention in older adults that members of the research group was working on. We found it adequate for an initial assessment of ChatGPT-4o’s capabilities in a focused and controlled setting, which we believe provided a sufficient foundation to identify potential patterns in performance and areas for improvement. | Discussion. Limitations section. |
| 2. I would be interested to know the agreement rate between the two authors? Suppose there was frequent disagreement between the two assessors, requiring a third author to intervene. Does this mean the data extracted by human standards was subjective and hence assessing ChatGPT against this subjective measure would have not shown its true capacity for extracting data from full-text papers? | We do not have any data on agreement rate for the data extraction of the source systematic review. For this study, the two authors had inserted the prompts independently, and compared the response of ChatGPT-4o to the reference standard. There was no disagreement from the authors assessing ChatGPT-responses, as the reference standard answers were clearly stated, and we simply assessed if these answers were the same or not in 5 prespecified categories, where the type of data extracted from the papers are objective information with little room for subjective influence. These mostly have one correct answer (e.g. mean age, gender proportion, number of dropouts).. While we acknowledge that some subjectivity may remain, this approach follows standard procedures in systematic reviews, where consensus-driven human extraction is used as the reference. We have added this to the discussion. | Discussion. Limitations section. |
| 3. How did you arrive at the prespecified assessment of the validity? Were there any previous similar studies using this model? | We designed the validity categories to reflect what we considered a fairly reliable and practical approach to evaluating ChatGPT-4o’s performance.  We have elaborated our explanation of the rationale for our prespecified categories. | Methods |
| 4. What was the prespecified goal of determining acceptable reproducibility? | We did not pre-specify goals for reproducibility, as we did for validity. Instead, we evaluated the results following the statistical analysis, which is a common practice for reproducibility assessments. Overall, we found the reproducibility to be generally high, with the exception of the outcome domain and cases where data was not reported. |  |
| 5. All figures and tables should have a legend summarising and explaining their results. All figures and tables from this study are missing legends. Graphs are also missing titles. | Thank you for your comments and feedback.  Legends and titles in the figures and tables are now included. | Figures and tables. |

Reviewer: #4

| **Reviewer comment** | **Author comment** | **Location of change in the manuscript** |
| --- | --- | --- |
| 1. The study notes that the questions were written without special knowledge of LLMs, which reflects an average user’s experience. However, exploring how optimized prompts could enhance AI performance might provide valuable insights. This could lead to recommendations on best practices for researchers using ChatGPT-4o. | We added to the discussion on how prompt engineering could be used to improve ChatGPT-4o’s data extraction accuracy and reliability**.** Specifically, we will outline how optimized prompts—designed to ask more specific, targeted questions or guide the model to recognize when data is missing—could mitigate errors and improve outcomes. | Discussion. From line 223. |
| 2. The study mentions that 5.2% of the data extracted by ChatGPT-4o was false, particularly when information was not reported in the papers. A deeper analysis of these errors—beyond reporting their frequency—could provide useful guidance on how to mitigate such risks. | We agree that a deeper analysis of the errors would provide valuable insights into how to mitigate the risk of false data extraction by ChatGPT-4o.  We have provided a more detailed discussion of the types of errors that occurred in the 5.2% of cases where ChatGPT-4o produced false information and offer potential reasons for why these mistakes may have occurred. | Discussion. From line 225. |
| 3. While the manuscript discusses the validity and reproducibility of ChatGPT-4o, it might benefit from a brief discussion on the ethical implications of using AI in systematic reviews, particularly regarding accountability and transparency when AI-generated data is integrated into research outputs | This is an intriguing and very relevant comment. We have added a discussion of the ethical implications of using AI in systematic reviews, particularly regarding accountability and transparency when AI-generated data is integrated into research outputs. | Discussion. From line 263. |
| 4. The study is based on a specific set of 11 RCTs focusing on fall prevention in older adults. While the methodology is robust, the generalizability to other fields or types of studies remains uncertain. What are the author's suggestions to expand the study to include a broader range of topics or study types that would strengthen the conclusions? | We acknowledge that the focus on a specific set of 11 RCTs may limit the generalizability of our findings to other fields or study types. However, the type of information processed in this study, such as publication details, participant characteristics, and outcomes, is not exclusive to fall prevention studies. These data categories are broadly applicable to other study types, including observational studies and non-RCT intervention studies.  In the revised manuscript, we suggest potential future research directions. Specifically, we propose expanding the use of ChatGPT-4o for data extraction across various types of systematic reviews, including studies with different research designs and topics, to assess the tool’s performance in more diverse contexts. | Discussion. From line 279. |
| 5. To add relevant information that supports the manuscript and benefits the readers, a suggested paper for investigating how ChatGPT can find and return real references. You may cite it as follows: <https://doi.org/10.1016/j.jormas.2024.101842>. | We appreciate your valuable feedback and will incorporate the reference to strengthen the manuscript. | Introduction. From line 63. |
